# Stage Migration in Cervical Cancer Using the FIGO 2018 Staging System: A Retrospective Survival Analysis Using a Single-Institution Patient Cohort

**DOI:** 10.7759/cureus.19289

**Published:** 2021-11-05

**Authors:** Toms Vengaloor Thomas, Kati K Reddy, Shivanthidevi Gandhi, Mary R Nittala, Anu Abraham, William Robinson, Mildred Ridgway, Satya Packianathan, Srinivasan Vijayakumar

**Affiliations:** 1 Radiation Oncology, University of Mississippi Medical Center, Jackson, USA; 2 Internal Medicine, University of Mississippi Medical Center, Jackson, USA; 3 Pathology, University of Mississippi Medical Center, Jackson, USA; 4 Obstetrics and Gynecology, University of Mississippi Medical Center, Jackson, USA

**Keywords:** figo, cervical cancer, radiation oncology, gynecology, obstetrics, stage, migration, oncology

## Abstract

Introduction

The International Federation of Gynecology and Obstetrics (FIGO) changed the staging system for cervical cancer in 2018 and formally allowed cross-sectional imaging for staging purposes. Stage IB is now divided into three substages based on tumor size (IB1 < 2 cm, IB2 2-4 cm and IB3 > 4 cm). The presence of lymph nodes in the pelvis or para-aortic region will upstage the patient to stage IIIC. The purpose of this study was to evaluate the extent of stage migration using the FIGO 2018 staging system for cervical cancer and validate the new staging system by assessing the survival outcomes.

Methods

An Institutional Review Board-approved and Health Insurance Portability and Accountability Act-compliant retrospective analysis was performed on 158 patients from the cervical cancer database at the University of Mississippi Medical Center, USA. Patients had been treated between January 2010 and December 2018, and they were all staged according to the FIGO 2009 staging system previously. We collected data regarding tumor size, lymph node presence, and extent of metastatic disease in the pretreatment CT, positron emission tomography (PET), or MRI scans and restaged the patients using the FIGO 2018 system. The extent of stage migration was evaluated using the new staging system. We analyzed the three-year overall survival (OS) using both FIGO 2009 and 2018 staging systems for validation purposes. Kaplan-Meier analyses were performed using SPSS version 24.

Results

Fifty-nine percent of the patients were upstaged when they were restaged using the FIGO 2018 staging system. In the current 2018 staging system, Stage IB3 accounted for 4%, and Stage IIIC accounted for 48% of the patient cohort, while other stages accounted for the rest. The median overall survival of the entire cohort was 20.5 months. There was a change in the survival curves using FIGO 2018 stages compared to those of FIGO 2009. There was a numerical improvement in three-year OS in stages IB and III among the two staging systems; however, it was not statistically significant. Interestingly, the three-year overall survival of Stage IIIC patients was better when compared to Stages III A& B combined (61% vs. 25%, p=0.017).

Conclusion

The increased availability of cross-sectional imaging across the world has led to recent changes in the FIGO staging system for cervical cancer, which allowed imaging in staging. We identified a significant stage migration in our patient cohort with the FIGO 2018 staging system, but no difference in the three-year overall survival was observed. Local tumor extent may be a worse prognostic indicator than nodal metastasis among stage III patients.

## Introduction

Cervical cancer is the fourth most common cancer in females worldwide [1-2]. It is the second most common cancer in women living in low- and middle-income countries and is a significant morbidity and mortality source [3]. Globally, there were approximately 569,847 new cases and 311,365 deaths reported in 2018 [1]. Over 85% of these cases are present in developing countries, where treatment and imaging resources are limited [4]. Cancer staging systems were designed to standardize the extent of anatomical disease at diagnosis or progression and compare patient outcomes across national boundaries [3]. Consequently, staging systems have to stay abreast of the development, distribution, and availability of advanced technology and the advent of new diagnostic and prognostic tools that could impact patient outcomes [3].

The International Federation of Gynecology and Obstetrics (FIGO) staging system for cervix cancer, previously revised in 2009, was updated again in 2018 to include the use of cross-sectional imaging modalities [3-4]. The modified 2018 FIGO staging system for cervical cancer allows cross-sectional imaging for staging purposes [4]. Stage IB is now divided into three instead of two sub-stages, based upon the tumor's size: IB1 < 2 cm, IB2 from two to 4 cm, and IB3 > 4 cm. Detection of lymph nodes in the pelvis or para-aortic region now upstages patients to IIIC [2-6]. Our intent in this analysis was to evaluate the extent of stage migration with the FIGO 2018 staging system for cervical cancer and retrospectively validate the new staging system using our institution’s patient cohort.

This work was previously presented as a poster at the American Society of Radiation Oncology (ASTRO) meeting in 2019. It was published in abstract form in the International Journal of Radiation Oncology, Biology, Physics on September 1, 2019 [7].

## Materials and methods

The necessary approvals were obtained from the University of Mississippi Medical Center (UMMC) Institutional Review Board (IRB) prior to the research. Due to the retrospective nature of the survival analyses, the written consent requirement was waived. We reviewed the records and charts data from the UMMC Cervical Cancer Database for patients diagnosed with and treated for cervical cancer between January 2010 and December 2018.

We identified 158 cervical cancer patients treated at UMMC during the study period. The patients had all been staged according to the FIGO 2009 staging system. We collected the data regarding tumor size, lymph nodes' presence, and extent of metastatic disease in the pretreatment CT, PET, or MRI scans.

Patients were then restaged using the FIGO 2018 system. We evaluated the extent of stage migration with the new staging system. We analyzed the three-year overall survival (OS) using both the FIGO 2009 and 2018 staging systems for validation purposes. We used SPSS version 24 software for data analysis. Kaplan-Meier analyses were used to evaluate survival parameters.

## Results

**Figure 1:**
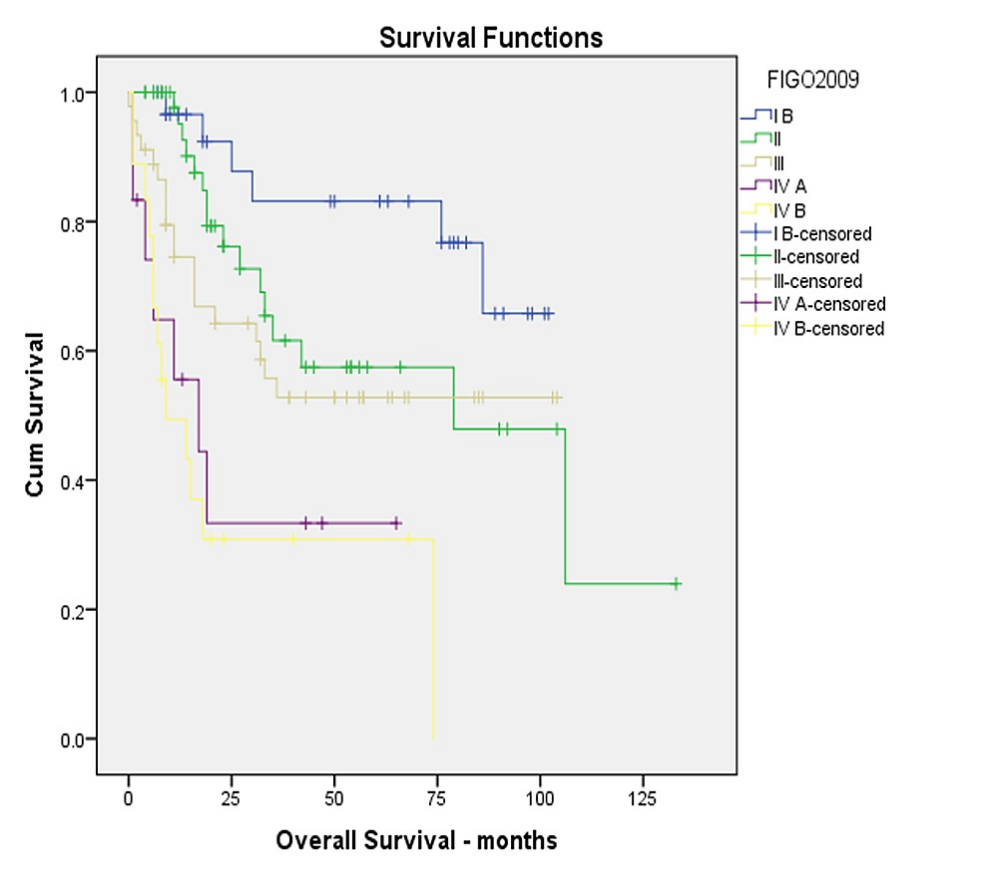
Kaplan-Meier overall survival using FIGO 2009 FIGO: International Federation of Gynecology and Obstetrics

**Figure 2:**
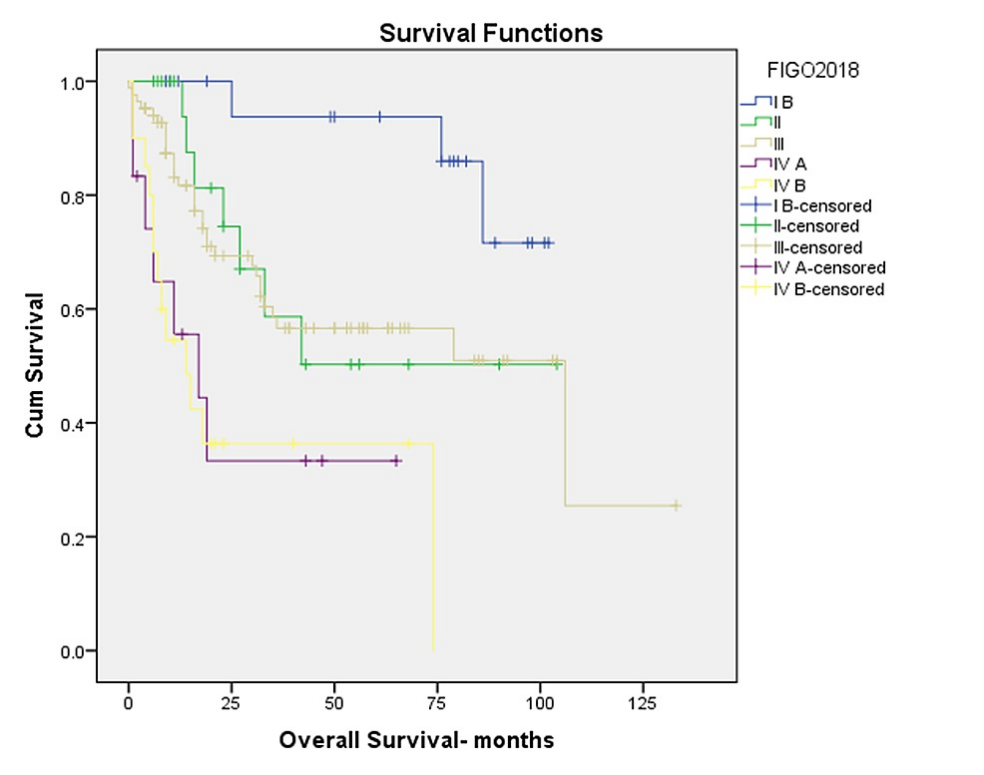
Kaplan-Meier overall survival using FIGO 2018 FIGO: International Federation of Gynecology and Obstetrics

We identified 158 cervical cancer patients treated during the study period. The FIGO 2018 staging system upstaged 59% of the patients. In the current 2018 staging system, Stage IB3 accounted for 4% and Stage IIIC accounted for 48% of the patient cohort, while other stages accounted for the rest. The median OS of the entire cohort was 20.5 months. There were changes observed in the survival curves in comparing FIGO 2018 to FIGO 2009, as depicted in Figures 1-2. There was a numerical improvement in three-year OS in stages IB and III among the two staging systems; however, it was not statistically significant (Table 1).

**Table 1:** Information on upstaging upon changing going from FIGO 2009 to FIGO 2018 FIGO: International Federation of Gynecology and Obstetrics

	FIGO 2009	FIGO 2009	FIGO 2018	FIGO 2018	
Stage	Frequency	3- year OS	Frequency	3-year OS	P-value
I B	31 (20%)	83%	21 (13%)	94%	0.4
II	52 (33 %)	62 %	21 (13%)	59 %	0.6
III	45 (28%)	53 %	84 (53%)	57%	0.6
IVA	12 (8 %)	33 %	12 (8%)	33%	1.0
IV B	18 (11%)	0	20 (13%)	0	
	158 (100%)		158 (100%)		

**Figure 3:**
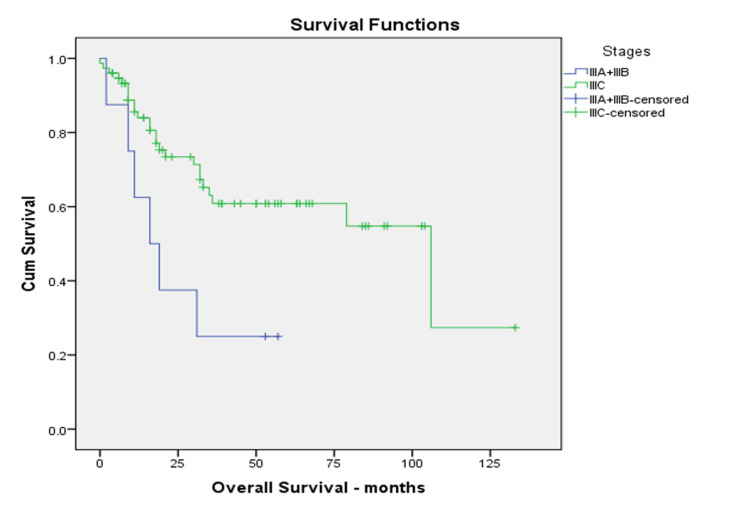
Kaplan-Meier overall survival for IIIA & B vs. IIIC

Interestingly, the three-year OS of Stage IIIC patients was better than Stages IIIA & B combined (61% vs. 25%, p=0.017), as depicted in Figure 3.

## Discussion

The present study was conducted to validate the new FIGO 2018 staging system by assessing the stage migration and survival outcomes. In the revised staging, Stage IB tumors are classified into three substages (IB1-IB3) based upon the tumor's size: IB1 < 2 cm, IB2 between 2 to 4 cm, and IB3 > 4 cm [2,8]. Patients with positive lymph nodes are classified as stage IIIC1 (pelvic lymph nodes) or IIIC2 (positive para-aortic nodes with or without pelvic lymph nodes) [2,9-10].

Stage migration

The process of restaging cervical cancer patients with the 2018 FIGO staging system has revealed some interesting distinctions within the stages. Yan et al. retrospectively reviewed 662 cervical cancer patients diagnosed at Zhejiang Cancer Hospital between 2008 and 2011 [11]. In this particular study, restaging using the FIGO 2018 staging system revealed an estimated 13% and 28% stage migration for Stage IB3 and Stage IIIC, respectively [11]. In an analysis of 251 cervical cancer patients conducted by Zeng et al., restaging the patients using the 2018 FIGO system resulted in an 11.2% and 7.2% stage migration for Stage IB3 and Stage IIIC, respectively [12]. Grigsby et al. reported that 53% of patients had stage migration when the FIGO 2018 system was used [13]. Tomizawa et al. noted that stage migration occurred in 53% of the patients from their single-institution study [14]. Our analysis identified that 59% of patients were upstaged: 4% to Stage IB3 and 48% to stage IIIC, with the other stages accounting for the rest when restaged using FIGO 2018. The stage migration seen when utilizing the new FIGO 2018 staging system in our analysis appears to be consistent with most of the literature on this subject.

Effect of imaging on stage migration in lymph node-positive patients

The incidence of para-aortic node (PAN) metastasis increases with the FIGO tumor stage [15-17]. For FIGO stages IB, II B, and III B disease, the risk of paraaortic node metastasis was about 5%, 20%, and 30%, respectively [14-16]. The presence of PANs was found to be the single most important independent predictor of relapse and survival in a multivariate analysis of 626 patients who were enrolled in Gynecologic Oncology Group (GOG) trials [18]. Grigsby et al. conducted a retrospective study of the patients staged with PET scans compared to CT alone [18]. In this study, the PET detected fluorodeoxyglucose (FDG)-avid metastatic disease in 21% compared with 7% in CT alone. Multivariate analysis showed that FDG-avid metastatic PAN by PET imaging is the most crucial predictor of progression-free survival. A follow-up prospective cohort study from the same institution confirmed the findings [19-20]. The PAN involvement's false-negative PET scan rates are around 12%, as evidenced by PAN status's surgical staging [19-20].

Survival outcomes 

The previously mentioned Yan et al. analysis found the FIGO 2018 stage to be an independent prognostic factor for OS [12]. In the Zeng et al. study mentioned before, the five-year progression-free survival rates showed the same trend with increasing stage reflecting upon the revised 2018 FIGO staging system's effectiveness in predicting outcomes [12]. On the other hand, in a retrospective study of 425 cervical cancer patients conducted by Ayhan et al., there were no significant differences in the five-year OS rates within the various sub-stages of IB disease [21]. Our analysis also did not find a significant difference in the three-year OS between the two staging systems.

Interestingly, our study revealed that Stage IIIC patients appear to have a better three-year OS than Stages IIIA and B combined (61% vs. 25%, p=0.017), consistent with many studies reported. In a retrospective study by Wright et al., higher FIGO staging did not consistently indicate worse five-year survival rates: stage IIIA (40.7%), stage IIIB (41.4%), stage IIIC1 (60.8%), and stage IIIC2 (37.5%) [9]. The Matsuo et al. validation analysis concluded that survival outcomes in Stage IIIC varied depending on the local tumor factors [6]. Furthermore, Liu et al.'s validation analysis concluded that the 2018 FIGO staging system does not consider the Stage IIIC local tumor and positive lymph node characteristics [5]. Some patients with low local stage (T stage) disease were upstaged to IIIC because of lymph node metastasis. In contrast, stages IIIA and B depend only upon the disease's local extent (T stage). Our findings suggest that patients with low volume local disease with nodal metastases might do better than those with high volume local disease without nodal metastases. Perhaps new sub-classifications of stage IIIC, which use the disease's local extent, may better differentiate these patients within the overall Stage IIIC rubric.

Future directions

Information on molecular factors that might affect cervical cancer outcomes may become an essential part of staging in the foreseeable future. The presence (or absence) of anti-angiogenic factors, such as VEGF-2, and immune checkpoint blockade markers, such as PD-L1, may significantly impact survival than either the local tumor or nodal metastasis [22]. They may also predict response to targeted agents such as bevacizumab, nivolumab, and pembrolizumab. Comprehensive, consistent testing for these and other markers will need to answer these questions.

Limitations

The two significant limitations to this study are the small patient cohort and the retrospective nature of this analysis. As this is a single institution’s retrospective analysis, some follow-up details were missing.

## Conclusions

The increased availability of cross-sectional imaging across the world has led to the permissibility of its use in the FIGO staging system for cervical cancer. A retrospective analysis of cervical cancer patients from January 2010 to December 2018 at a major academic medical center identified a significant degree of stage migration in the patient cohort using the FIGO 2018 staging system. However, our study found that FIGO 2018 staging was not superior in predicting three-year OS than FIGO 2009. Local tumor extent may be a worse prognostic indicator than nodal metastasis among stage III patients. A new sub-classifications of stage IIIC, which uses the disease's local extent, may better differentiate these patients within the overall Stage IIIC. The incorporation of imaging findings into FIGO staging has improved physicians’ ability to stage the patients better and strategize the treatment. Further refinement of the staging systems is warranted with the improvement in imaging capabilities.

## References

[1] Bray F, Ferlay J, Soerjomataram I, Siegel RL, Torre LA, Jemal A (2018). Global cancer statistics 2018: GLOBOCAN estimates of incidence and mortality worldwide for 36 cancers in 185 countries. CA Cancer J Clin.

[2] Bhatla N, Aoki D, Sharma DN, Sankaranarayanan R (2018). Cancer of the cervix uteri. Int J Gynaecol Obstet.

[3] Bhatla N, Berek JS, Cuello Fredes M (2019). Revised FIGO staging for carcinoma of the cervix uteri. Int J Gynaecol Obstet.

[4] Jolly S, Uppal S, Bhatla N, Johnston C, Maturen K (2018). Improving global outcomes in cervical cancer: the time has come for International Federation of Gynecology and Obstetrics Staging to formally incorporate advanced imaging. J Glob Oncol.

[5] Liu X, Wang J, Hu K (2020). Validation of the 2018 FIGO Staging System of Cervical Cancer for Stage III patients with a cohort from China. Cancer Manag Res.

[6] Matsuo K, Machida H, Mandelbaum RS, Konishi I, Mikami M (2019). Validation of the 2018 FIGO cervical cancer staging system. Gynecol Oncol.

[7] Thomas TV, Gandhi S, Nittala M (2019). Stage migration in cervical cancer using the FIGO 2018 staging system: a retrospective survival analysis using a single institution patient cohort. Is there a Will Rogers phenomenon?. Int J Radiat Oncol Biol Phys.

[8] Sehnal B, Kmoníčková E, Sláma J, Tomancová V, Zikán M (2019). Current FIGO staging for carcinoma of the cervix uteri and treatment of particular stages. Klin Onkol.

[9] Wright JD, Matsuo K, Huang Y (2019). Prognostic performance of the 2018 International Federation of Gynecology and Obstetrics Cervical Cancer staging guidelines. Obstet Gynecol.

[10] Horn LC, Brambs CE, Opitz S, Ulrich UA, Höhn AK (2019). The 2019 FIGO classification for cervical carcinoma—what's new? [Article in German]. Pathologe.

[11] Yan DD, Tang Q, Chen JH, Tu YQ, Lv XJ (2019). Prognostic value of the 2018 FIGO staging system for cervical cancer patients with surgical risk factors. Cancer Manag Res.

[12] Zeng J, Qu P, Hu Y, Sun P, Qi J, Zhao G, Gao Y (2020). Clinicopathological risk factors in the light of the revised 2018 International Federation of Gynecology and Obstetrics staging system for early cervical cancer with staging IB. A single center retrospective study. Medicine (Baltimore).

[13] Grigsby PW, Massad LS, Mutch DG (2020). FIGO 2018 staging criteria for cervical cancer: impact on stage migration and survival. Gynecol Oncol.

[14] Tomizawa K, Kaminuma T, Murata K (2020). FIGO 2018 staging for cervical cancer: influence on stage distribution and outcomes in the 3D-image-guided brachytherapy era. Cancers (Basel).

[15] Berman ML, Keys H, Creasman W, DiSaia P, Bundy B, Blessing J (1984). Survival and patterns of recurrence in cervical cancer metastatic to periaortic lymph nodes. A Gynecologic Oncology Group study. Gynecol Oncol.

[16] Delgado G, Bundy BN, Fowler WC (1989). A prospective surgical pathological study of stage I squamous carcinoma of the cervix: a Gynecologic Oncology Group Study. Gynecol Oncol.

[17] Lagasse LD, Creasman WT, Shingleton HM, Ford JH, Blessing JA (1980). Results and complications of operative staging in cervical cancer: experience of the Gynecologic Oncology Group. Gynecol Oncol.

[18] Stehman FB, Bundy BN, DiSaia PJ, Keys HM, Larson JE, Fowler WC (1991). Carcinoma of the cervix treated with radiation therapy I. A multi-variate analysis of prognostic variables in the Gynecologic Oncology Group. Cancer.

[19] Gouy S, Morice P, Narducci F (2012). Nodal-staging surgery for locally advanced cervical cancer in the era of PET. Lancet Oncol.

[20] Gouy S, Morice P, Narducci F (2013). Prospective multicenter study evaluating the survival of patients with locally advanced cervical cancer undergoing laparoscopic para-aortic lymphadenectomy before chemoradiotherapy in the era of positron emission tomography imaging. J Clin Oncol.

[21] Ayhan A, Aslan K, Bulut AN, Akilli H, Öz M, Haberal A, Meydanli MM (2019). Is the revised 2018 FIGO staging system for cervical cancer more prognostic than the 2009 FIGO staging system for women previously staged as IB disease?. Eur J Obstet Gynecol Reprod Biol.

[22] Minion LE, Tewari KS (2018). Cervical cancer - state of the science: from angiogenesis blockade to checkpoint inhibition. Gynecol Oncol.

